# Evidence of Multifidus Changes Post-Lumbar Radiofrequency Ablation: A Narrative Literature Review

**DOI:** 10.3390/jcm14186462

**Published:** 2025-09-13

**Authors:** Abigail Joy Garcia, David W. Lee, Logan Leavitt, Vinicius Tieppo Francio

**Affiliations:** 1 Department of Physical Medicine & Rehabilitation, University of Texas Health Science Center San Antonio, 7703 Floyd Curl Dr., San Antonio, TX 78229, USA; 2 University of California Irvine, Fullerton Orthopedics, Fullerton, CA 92834, USA; 3 Barrow Brain and Spine, 4530 Muirwood Drive STE 110, Ahwatukee, AZ 85048, USA; 4 Division of Pain Medicine, Department of Anesthesiology, Washington University School of Medicine in St. Louis, 4921 Parkview Place, 14th Floor, Suite C, St. Louis, MO 63110, USA

**Keywords:** low back pain, lumbar radiofrequency ablation, lumbar rhizotomy, multifidus muscle, multifidus atrophy, multifidus dysfunction

## Abstract

**Background/Objectives**: Chronic low back pain (CLBP) is a leading cause of disability worldwide, with lumbar medial branch radiofrequency ablation (LRFA) widely used to manage facet-mediated pain; however, emerging evidence raises concerns regarding its potential to denervate the multifidus muscle—an essential stabilizer of the lumbar spine—thereby exacerbating dysfunction. This narrative review synthesizes current evidence on multifidus atrophy and dysfunction following LRFA, emphasizes its clinical significance, and highlights gaps that warrant further research and therapeutic development. **Methods**: A comprehensive literature search was conducted using SANRA criteria across the Cochrane Library, Web of Science Core Collection, Scopus, PubMed, and MEDLINE. Studies assessing multifidus morphology or function after LRFA were identified and analyzed. Data were extracted from studies meeting predefined inclusion criteria. The narrative synthesis included a thematic analysis and interpretive integration focusing on clinical practice. **Results**: Six eligible studies were identified, five cohort studies and one case series. Of these, two confirmed decreased multifidus function post-LRFA. Four studies analyzed post-LRFA structural changes, two of which reported reduced cross-sectional area/fatty infiltration, one no measurable difference, and another an apparent enlargement. The findings are constrained by substantial differences in study design, patient populations, and outcome measures, which limit the ability to establish consistent conclusions. **Conclusions**: Current evidence suggests that LRFA may lead to structural and functional changes in the multifidus muscle, although findings remain inconsistent due to significant study heterogeneity. Further high-quality, prospective research with standardized imaging and functional assessments is needed to clarify the long-term clinical impact.

## 1. Introduction

Chronic low back pain (CLBP) represents a significant public health challenge due to its high prevalence and substantial impact on patient quality of life and functional capacity [1]. Among the various etiologies of CLBP, facet joint-mediated pain represents a significant contributor, with reported prevalence rates ranging from approximately 4.8% to over 50% [2,3]. This wide prevalence range largely stems from a varying level of stringent diagnostic and patient selection criteria in studies assessing the prevalence of facet-mediated pain, with studies that have more stringent criteria resulting in lower prevalence rates [4]. The lumbar facet joints receive their primary innervation from the lumbar medial branch nerves (LMBN). Each facet joint has dual innervation stemming from the LMBN at the corresponding vertebral level and the vertebral level immediately above [5,6].

Lumbar medial branch radiofrequency ablation (LRFA), first introduced by Dr. Nikolai Bogduk in 1980, remains a widely used interventional treatment for facet-mediated CLBP [7,8]. LRFA ranks as the second most frequently performed intervention for facetogenic CLBP, underscoring its pivotal role in clinical practice [9]. The mechanism of action involves thermal ablation of the LMBN, effectively disrupting afferent nociceptive transmission from the facet joints to the spinal dorsal horn, thereby providing pain relief [10]. There remains heterogeneity amongst research studies regarding the long-term effectiveness of LRFAs. Some studies have shown an improvement in Oswestry Disability Index, quality of life, and pain scores following LRFA, while others demonstrate no clinically significant improvement in individuals who received an LRFA compared to an exercise program alone or placebo [11,12,13,14]. Furthermore, a Cochrane review found that there is high-quality evidence that the procedure provides pain relief; however, evidence is lacking that it improves function [15]. A potential reason for the lack of evidence in improving function is the impact of LRFA on paraspinal muscle structure and function, particularly the multifidus muscle, which remains a subject of ongoing debate. This controversy arises from the anatomical overlap between the LMBN mutual innervation of the lumbar facet joint (afferent nociceptive fibers) and the multifidus muscle (efferent motor fibers), raising concerns about unintended multifidus denervation and subsequent multifidus dysfunction and atrophy [5,16].

Understanding the clinical implications of multifidus denervation is critical as these muscles are essential stabilizers of the lumbar spine, responding dynamically to movement and postural adjustments [17,18]. Multifidus dysfunction has been extensively documented as a key contributor to CLBP, primarily through muscle inhibition, dysfunction, and the progressive loss of neuromuscular feedback and spinal stability— factors that collectively exacerbate pain and further impair function [19,20,21,22].

Given this backdrop, the objectives of this narrative review are to synthesize existing evidence on multifidus atrophy and dysfunction following LRFA, discuss the clinical relevance of these findings, and identify possible areas requiring further research and treatment gaps on the sequelae of LRFA.

## 2. Materials and Methods

**Figure 1:**
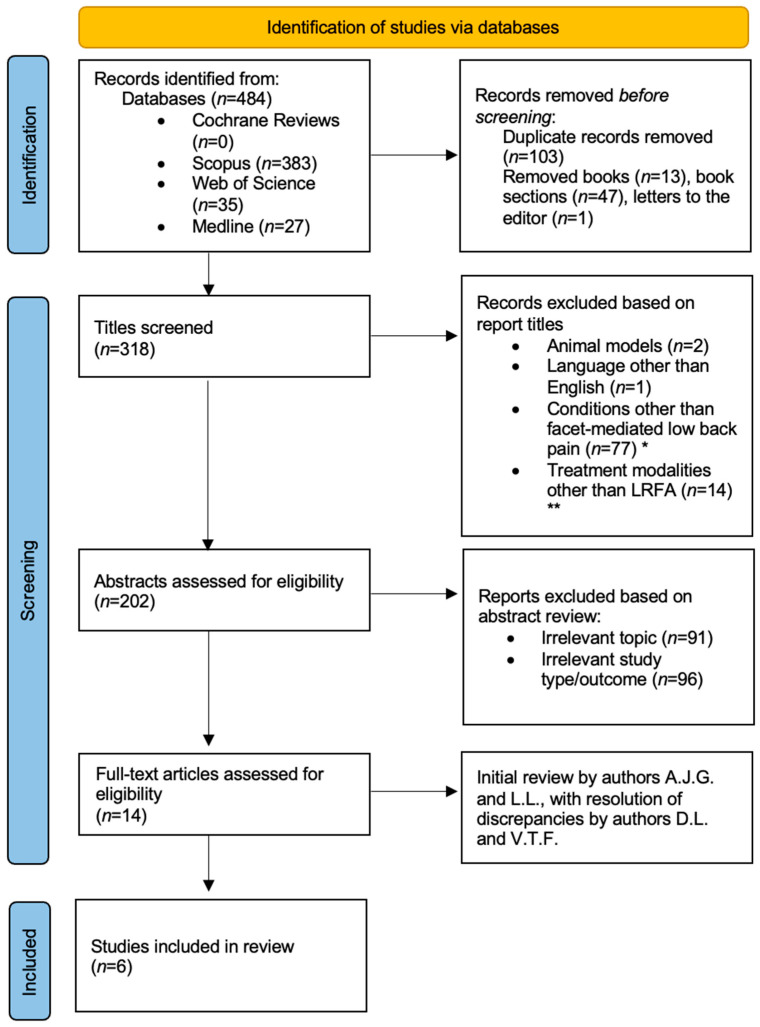
Modified PRISMA diagram for narrative literature review. * Conditions other than facet-mediated low back pain included cervical/cervicogenic pain (*n* = 20), whiplash (*n* = 8), cancer/metastatic disease/tumors (*n* = 10), disc herniations/discogenic pain (*n* = 26), sacroiliac joint pain and dysfunction (*n* = 9), vertebral fractures including spondylolysis (*n* = 4). ** Treatment modalities other than LRFA included epidural steroid injections (*n* = 4), endoscopic medial branch rhizotomy (*n* = 2), dorsal root ganglion block (*n* = 1), surgical interventions or post-surgical individuals (*n* = 17). This diagram was generated from a PRISMA template [24].

This narrative review was conducted in accordance with established best practices for narrative synthesis, emphasizing transparency, methodological rigor, and interpretive depth as outlined by Sukhera et al. [23]. This review aimed to synthesize and contextualize the current evidence regarding multifidus atrophy or dysfunction following LRFA, while also exploring gaps that warrant further research and therapeutic development within the landscape of CLBP. We began by clearly defining the scope, rationale, and clinical relevance of this review. A comprehensive literature search was conducted across the Cochrane Library, Web of Science Core Collection, Scopus, PubMed, and MEDLINE between January and May 2025. Search terms used were “multifidus” AND “radiofrequency,” with results limited from 2000 to 2025. These search criteria elucidated 484 results. The reference tool, Zotero, was used to aid in systematic screening. After removing duplicates, 381 studies remained. Eligibility criteria were established a priori (Table 1), based on study design, population, and relevance to the research questions. Inclusion was limited to peer-reviewed human studies published in English, encompassing randomized controlled trials, observational studies, and case series. Results were narrowed by evaluating titles and excluding conditions that did not involve the facet joints, as well as treatment modalities that were not radiofrequency ablations to the medial branch (Figure 1). Out of the remaining 205 studies, the authors reviewed abstracts to further narrow the focus to include those that specifically evaluated multifidus atrophy after LRFA(s), resulting in 14 studies. The full texts were reviewed for these final 14 studies by all authors to reach a consensus. In situations where conflicts of interest were identified, authors were instructed to abstain from decision-making.

Data from eligible studies were extracted using a standardized template that included study design, sample characteristics, methods of assessing multifidus structure or function, key outcomes, and author interpretations. In line with narrative review methodology, data synthesis employed both descriptive (e.g., frequency of study designs and reported outcomes) and interpretive (e.g., thematic and clinical implications) approaches. Thematic patterns were identified and reported in the Results and Discussion Sections. Throughout the review process, the authors engaged in reflexive deliberation, acknowledging how their clinical and academic perspectives shaped study selection, interpretation, and framing. This review adheres to the SANRA (Scale for the Assessment of Narrative Review Articles) criteria, which assess the methodological quality of narrative reviews across six domains: topic significance, clarity of aims, adequacy of the literature search, referencing quality, strength of scientific reasoning, and appropriateness of data presentation [25]. These principles guided the development, execution, and reporting of this review. Finally, consistent with the conventions of rigorous narrative synthesis, we explicitly recognize key limitations, including the potential non-exhaustiveness of the search strategy, the possibility of selection and interpretation bias, and the limited generalizability of findings across clinical contexts.

## 3. Results

A total of six studies evaluating structural or functional changes in the multifidus muscle following LRFA were identified, including five cohort studies and one case series. Study designs varied in methodology, with sample sizes ranging from 1 to 46 patients. Among the cohort studies, most utilized T2-weighted MRI of the lumbar spine to assess outcomes, while others employed advanced imaging techniques such as fat-subtracted imaging, shear-wave elastography, and electromyography (Table 2).

## 4. Discussion

### 4.1. Multifidus Atrophy Following LRFA

While LRFA is generally regarded as a safe procedure, the potential issue of post-LRFA multifidus denervation remains controversial [32]. A key contributor to this ongoing debate is the scarcity of high-quality studies specifically evaluating multifidus atrophy. Moreover, the studies identified in our search and included in this review have substantial variability in design, measurement methods, and follow-up periods. Most of the studies enrolled fewer than thirty subjects, resulting in limited statistical power and reduced generalizability of findings. This heterogeneity considerably limits comparability and undermines the ability to draw robust conclusions. Notably, most of the studies included do not link the imaging findings with functional or clinical outcomes, highlighting the critical need for future research that includes these aspects to enhance the relevance to clinical practice. Addressing this gap, a recent systematic review critically examined the literature on structural and functional changes in the multifidus muscle following LRFA [16]. The authors found three Level IB and two Level IC studies evaluating this potential relationship; however, these studies were of poor-to-fair quality and demonstrated a very low level of certainty based on the GRADE criteria. Given the nature of LRFA and its inherent denervation mechanism, some degree of multifidus atrophy is biologically plausible, and it has been confirmed through electromyography within six weeks of the procedure and further supported by shear-wave elastography findings. Importantly, these observations suggest that functional alterations may occur early and could precede structural changes detectable on MRI [16].

Given the fragmented nature of existing studies and the lack of high-quality evidence, a comprehensive synthesis of available data is essential to better understand the potential sequelae of LRFA on the multifidus. This review builds upon prior systematic analyses by integrating newly published studies, critically examining individual study findings and their clinical implications, and highlighting persistent knowledge gaps that warrant further research and the development of novel therapeutic strategies within the broader management algorithm of CLBP and post-LRFA multifidus atrophy.

The results of Dreyfuss et al. and Böning et al. should be interpreted with caution because of severe limitations in the study designs. Dreyfuss’ study was one of the first published on LRFA and multifidus atrophy. Dreyfuss et al. concluded that LRFA does not cause segmental atrophy of the multifidus at long-term follow-up [31]; however, the study has several notable flaws. Most critically, there was no comparison of pre-procedure and post-procedure MRI scans. Instead, blinded radiologists were asked to assess only post-procedure MRIs and determine the side treated by LRFA based on the amount of atrophy observed. More accurately, the radiologists’ decisions should be based on the change in the multifidus muscle. Additionally, the study included a very limited sample size of only five participants, with variable follow-up intervals, and lacked demographic information such as gender, age, and relevant comorbidities. Collectively, these limitations undermine the strength and generalizability of the study findings.

The primary objective of Böning et al. was to assess the feasibility of performing LRFA under open MRI guidance rather than using conventional C-arm fluoroscopy [28]. To complement their analysis of pain scores, the authors manually measured multifidus volume using 3D program software that generates a model of the multifidus based on axial T2-weighted MRI images of the multifidus one week before LRFA and six months post-LRFA. The authors conclude that the volume of the multifidus increased following the procedure, which they attributed to pain relief reversing hypotrophy of the muscle. However, they did not differentiate the proportion of true skeletal muscle versus fat infiltration within the measured volume. Consequently, these findings should be approached with caution as it is unclear what contribution fat infiltration and atrophy had in this volumetric increase.

Sadeghi et al. used ultrasound shear-wave elastography to evaluate the shear modulus of the multifidus after LRFA, with a higher shear modulus reflecting greater tissue stiffness [29,33]. Measurements in the multifidus shear modulus were obtained within two years post-LRFA in three positions: prone, seated, and seated with arms lifted horizontally. Compared with healthy controls, the post-LRFA multifidus demonstrated no increase in shear modulus across these positions, suggesting dysfunction and impaired contractility consistent with LMBN denervation. One potential explanation for the reduced change in shear modulus is fatty infiltration and replacement of muscle fibers with non-contractile fibrous tissue in the setting of atrophy. However, shear-wave elastography cannot distinguish whether this decrease resulted from denervation-related dysfunction or true structural atrophy. Notably, it is well-established that LRFA denervates the multifidus, inhibiting its ability to contract, and prior studies have confirmed successful LRFA by demonstrating absent multifidus motor activity on EMG [31,34]. Thus, while it is a fair-quality study, the findings of Sadeghi et al. do not clarify whether multifidus atrophy occurs post-LRFA.

Three of the six studies utilized MRI to investigate multifidus atrophy. Smuck et al., Oswald et al., and Guven et al. utilized pre- and post-LRFA MRI imaging to assess changes in multifidus morphology [26,27,30]. Smuck used a gray-scale measurement to determine the amount of fat infiltration in the multifidus, which they refer to as the fat-subtracted cross-sectional area [30]. Oswald and Guven used different computer software programs to three-dimensionally measure the relative muscle and fat volumes of the multifidus. Despite methodological differences, both Smuck and Guven concluded that there was a reduced skeletal muscle cross-sectional area following LRFA, although Smuck’s study did not reach statistical significance. Conversely, Oswald concluded the opposite—that the relative intramuscular fat volume of the multifidus did not change post-LRFA [27]. There are several potential reasons as to why these studies may have reached variable conclusions. Each study involved varying follow-up periods for repeated imaging. Oswald had a minimum of 6 months, Smuck had an average of 7.5 months, and Guven had a minimum of 2 years. This raises the important question of when multifidus atrophy should be expected status-post LRFA. Studies show that molecular and biochemical changes begin almost immediately following nerve denervation, occurring through a decline in protein synthesis and elevated proteolysis. While histological change and functional decline are noted within a few weeks, radiographic and gross atrophy may not be identifiable for at least a few months.

Furthermore, each study differed in how many repeat LRFAs their patients received; variations in the procedure being performed bilaterally or unilaterally; and the number of levels treated. Guven included patients who received only 1 unilateral LRFA, whereas Smuck included patients who received 1–3 repeated LRFAs. Oswald included both unilateral and bilateral LRFAs but they were only performed one time. This raises the question of whether multifidus atrophy is more of a risk that may occur upon receiving repeat LRFAs. Notably, all three studies were limited by small sample sizes (Oswald: *n* = 20; Guven: *n* = 24; Smuck: *n* = 27) and retrospective designs, both of which reduce statistical power and introduce potential sources of bias. Each of these three retrospective studies included subjects who received repeated imaging following LRFA, which may inadvertently select for individuals who have worsening symptoms or progressive degeneration, thereby limiting generalizability. Additionally, the choice of control conditions varied across studies. Smuck et al. used untreated segmental levels within the same patient as controls, whereas Oswald used the contralateral side as a control in unilateral LRFA cases but lacked controls for bilateral procedures. These control choices are potentially confounding as it is still debated whether the multifidus muscle is monosegmentally or polysegmentally innervated. Anatomical studies suggest monosegmental innervation but electromyographic evidence indicates polysegmental innervation [35,36]. Thus, denervation at one level could potentially influence the morphology of adjacent-level multifidi, and conversely, multifidus atrophy may not occur if adjacent medial branches are not ablated.

Given the relatively high methodological quality of the studies by Smuck et al. and Oswald et al., as previously assessed by Tieppo Francio et al. using the Newcastle–Ottawa Scale, their conclusions may be regarded with greater confidence [16]. Nevertheless, variability in the study design, imaging methodologies, sample size, retrospective methods—coupled with conflicting results—leaves the question of the presence and extent of post-LRFA multifidus atrophy unresolved. The scarcity of high-quality research and the lack of standardized methods to evaluate post-procedural structural and functional changes underscore the need for rigorous, well-designed prospective studies.

Three additional studies contribute useful observations to this review. While their contributions are limited by the absence of generalizability and lack of controlled methodology, they collectively suggest a possible association between LRFA and multifidus changes. For example, Vas et al. report a case involving a 65-year-old woman who exhibited worsened bilateral multifidus atrophy on T2-weighted MRI following bilateral LRFA [37]; however, the authors did not specify the interval between the procedure and the follow-up MRI.

Bajaj et al. reported a case of dropped head syndrome in a 77-year-old patient following staged bilateral cervical RFA of the C3–C5 medial branches [38]. Six weeks post-RFA, the patient developed marked cervical flexion and paraspinal weakness, with only partial recovery after ten weeks of physical therapy. While this case lacks pre- or post-RFA imaging, it highlights the potential for delayed neuromuscular deficits following cervical RFA, although the precise mechanisms remain uncertain. This highlights the importance of assessing clinical outcomes in future investigations of post-LRFA multifidus atrophy, in addition to the muscle morphology. Finally, Almalki et al. used validated computational modeling to simulate unilateral and bilateral LRFA by reducing the multifidus cross-sectional area and force generation [39]. The model demonstrated altered spinal biomechanics during flexion, lateral bending, and rotation, with increased joint angles that may predispose to accelerated disc degeneration, reflecting the loss of multifidus stabilization. Although the study was an in vivo study, its findings align with those of Guven et al. and Smuck et al., who demonstrated structural atrophy and fatty infiltration of the multifidus following LRFA.

### 4.2. Clinical Implications

The lumbar multifidus muscle plays a pivotal role in maintaining dynamic stability of the spine, contributing to approximately two-thirds of the lumbar spine’s stiffness in a neutral position [40]. Characterized by a large physiological cross-sectional area and short muscle fiber length, the multifidus is biomechanically optimized to generate substantial force over a limited range of motion. Its predominance of type I muscle fibers further supports its primary function as a tonic stabilizer. Given this critical biomechanical role, lumbar multifidus dysfunction has been strongly implicated in the pathophysiology of CLBP [19,41].

Abdelaty et al. demonstrated that patients with chronic non-specific LBP exhibited impaired voluntary activation of the multifidus muscle compared to healthy controls [42]. This neuromuscular dysregulation leads to reduced muscle recruitment and diminished contraction strength, ultimately compromising segmental spinal stability. Persistent hypoactivity promotes disuse atrophy, further limiting functional engagement of the muscle and perpetuating a cycle of instability, pain, and mechanical deconditioning [42]. The adverse consequences of multifidus atrophy or dysfunction on spinal biomechanics, as well as their contribution to the chronicity and recurrence of low back pain, have been well-documented in the recent literature [19].

Emerging evidence has further elucidated the multifidus muscle’s involvement in broader spinal pathologies. For instance, multifidus atrophy has been associated with lumbar facet joint osteoarthritis and isthmic spondylolisthesis, with the degree of vertebral slippage positively correlating with the extent of muscular degeneration [43]. Additionally, there is a close anatomical and functional interplay between the multifidus and the intervertebral disc, with studies demonstrating a strong association between multifidus atrophy and disc degeneration [44]. Recent investigations have also explored the molecular and metabolic mechanisms of fat infiltration in the multifidus. Immune-mediated mechanisms and disruptions in lipid metabolism appear to drive this process. For example, Chen et al. reported that patients with lumbar disc herniation and severe multifidus fat infiltration exhibited a 28-fold increase in TNF-α expression compared to those with minimal fatty infiltration [45]. TNF-α activates the NF-κB signaling pathway, accelerating extracellular matrix breakdown, promoting cellular apoptosis, and contributing to progressive disc degeneration [45]. Given concerns regarding potential multifidus atrophy following LRFA, alternative therapeutic strategies for chronic low back pain (CLBP) that stimulate, rather than ablate, the lumbar medial branch nerve (LMBN) have garnered increasing attention. Unlike LRFA, which achieves analgesia through denervation of the LMBN, restorative neurostimulation applies targeted bilateral electrical stimulation to the L2 LMBN with the aim of reactivating the multifidus muscle and restoring its functional control. Emerging evidence from prospective studies demonstrates promising longitudinal outcomes, with reports of sustained improvements in pain, function, and quality of life, as well as reduced healthcare utilization over follow-up periods extending up to five years, including superiority over standard conservative management at one year based on RCTs and real-world studies [46,47,48,49,50,51,52,53,54,55,56,57].

## 5. Limitations

This review is subject to several important limitations. First, there is considerable heterogeneity across the included studies in terms of patient selection criteria, sample sizes, and outcome measures, which complicates efforts to draw consistent conclusions or generalize findings to broader clinical populations. Second, many of the existing studies are limited by relatively short follow-up durations, making it difficult to assess the long-term structural and functional consequences of multifidus denervation following LRFA. Finally, there remains a critical need for prospective, high-quality trials that incorporate standardized imaging protocols alongside objective functional testing to more precisely characterize the temporal evolution of multifidus changes post-LRFA and clarify their relationship to clinical outcomes.

## 6. Conclusions

Emerging evidence suggests that LRFA may cause multifidus denervation, with functional changes demonstrated in two studies and structural atrophy reported in two others; however, the overall evidence remains limited as most available studies are of poor-to-fair quality and provide low-certainty findings, limiting their generalizability. Once considered a theoretical concern, multifidus atrophy and dysfunction are now increasingly recognized as clinically meaningful contributors to chronic low back pain. In parallel, novel therapeutic strategies aimed at reactivating the multifidus through restorative neurostimulation have shown promising long-term improvements in pain, function, and quality of life. These findings highlight the importance of balancing the potential benefits of LRFA with its possible long-term effects on muscle function. Larger, high-quality, prospective studies using standardized imaging and functional assessments are needed.

## Figures and Tables

**Table 1:** Inclusion and exclusion criteria for the narrative review.

Inclusion Criteria	Exclusion Criteria
Original peer-reviewed research	Book chapters, letters to the editor, conference abstracts
English language	Manuscripts in languages other than English
Human studies	Non-human studies
Study designs: randomized controlled trials, prospective/retrospective observational cohorts, case series, case reports	Duplicate data
Outcomes assessing multifidus morphology or function post-LRFA	

**Table 2:** Baseline characteristics of the included studies.

Author	Study Design	Number of Subjects	Outcome Tool Measurement (Multifidus Measurement Method)	Measurement Timing	Follow-Up Intervals After LRFA
Guven [26]	Retrospective longitudinal case series with paired analysis	24 patients (51 spinal levels)	Custom software analysis of multifidus fat area based on T2w MRIs	Pre- and post-LRFA	Over 2 years
Oswald [27]	Retrospective, longitudinal cohort study	20	Semi-automatic analysis of standard T2w MRI of L-spine	Pre- and post-LRFA	16.8 months (median)
Böning [28]	Prospective, longitudinal cohort study	17	Manual measurement of axial T2w MRI of L-spine	Pre- and post-LRFA	1 week and 6 months
Sadeghi [29]	Prospective, cross-sectional cohort study	46	Customized supersonic ultrasound SWE of multifidus at the middle level	Post-LRFA	11.42 months (mean)
Smuck [30]	Retrospective, longitudinal, cohort study	27	Fat-subtracted multifidus CSA with gray-scale cutoff values of axial T2w MRI of L-spine	Pre- and post-LRFA	7.5 months (mean)
Dreyfuss [31]	Prospective, cross-sectional cohort study	5	T2w sagittal and T1w axial MRI images of L-spine and EMG	Post-LRFA	21 months (mean)

CSA = cross-sectional area; EMG = electromyography; L-spine = lumbar spine; LRFA = lumbar radiofrequency ablation; MRI = magnetic resonance imaging; SWE = shear-wave elastography; T1w = T1-weighted; T2w = T2-weighted.

## Data Availability

No new data were created or analyzed in this study. Data sharing is not applicable to this article.

## Abbreviations

The following abbreviations are used in this manuscript:

CLBP	Chronic low back pain
LMBN	Lumbar medial branch nerves
LRFA	Lumbar medial branch radiofrequency ablation
SANRA	Scale for the Assessment of Narrative Review Articles
EMG	Electromyography
MRI	Magnetic resonance imaging
FCSA	Functional cross-sectional area
PNS	Peripheral nerve stimulation
VAS	Visual analog scale
ODI	Oswestry Disability Index
ASPN	American Society of Pain and Neuroscience
ISASS	International Society for the Advancement of Spine Surgery
